# Commentary: Sensitivity, Specificity, and Predictive Values: Foundations, Pliabilities, and Pitfalls in Research and Practice

**DOI:** 10.3389/fpubh.2018.00256

**Published:** 2018-09-28

**Authors:** Gilat Grunau, Shai Linn

**Affiliations:** ^1^Department of Radiology, Vancouver General Hospital, University of British Columbia, Vancouver, BC, Canada; ^2^School of Public Health, University of Haifa, Haifa, Israel

**Keywords:** Bayes' theorem, clinical, diagnosis, epidemiology, methods, screening

We would like to suggest new perspectives, following Trevethan's article (1), on misconceptions about the measures of screening.

## Measures of test accuracy in two distinct situations: detection and diagnosis (2–20)

We suggest a clear distinction between the accuracy measures of the **detection of a disease in a screening setting in populations**, using data on persons with and without known disease status, vs. accuracy measures of **diagnosis in a clinical setting** in individuals when the disease status is unknown.

In the first situation, a researcher is conducting a study in which the prevalence of the disease is **artificial:** it is determined by the researcher based on the number of persons with and without the disease who are included in the study. For example, if in a study one examines the *sensitivity* and *specificity* of a test in 100 persons with a disease (for example, AIDS) and 100 persons without the disease, the prevalence of AIDS in this particular study is 50%, which is of course far from the true prevalence of AIDS. The *sensitivity* and *specificity* are used to describe the *technical* characteristics of a test, i.e., how many persons with a disease or without a disease will be **detected correctly** by a test in a population with known diagnoses. These measures are important in public health and health planning. For example, one might need to know the percentage of sick persons (the *sensitivity*) or healthy persons (the *specificity*) that will be detected among travelers at an airport and thus plan preventive measures in times of a communicable disease epidemic.

The second situation is a clinical setting when the diagnoses are as yet unknown and the test is used to diagnose the disease in individuals: the positive predictive value (PPV) and the negative predictive value (NPV) are an estimate of the accuracy of the test, i.e., of the fractions of patients who are **diagnosed correctly** as positive or negative, respectively.

Let us explain the need to use two different approaches for the two distinct situations described above, with specific analogous notations (italic lowercase and uppercase) and the derived equations.

### Estimating the accuracy of test detectability in populations (Table 1)

We use italic **lower-case letters** in the description of screening in the general population in a 2 × 2 table, Table 1. The *sensitivity* and *specificity* are calculated in samples of persons with (a + c) and without (b + d) the disease in a selected population. In this table, it is inappropriate to include totals of the “horizontal” axis of test (T) results.

**Table 1:** Data presentation in a selected population, assessing detectability of a test.

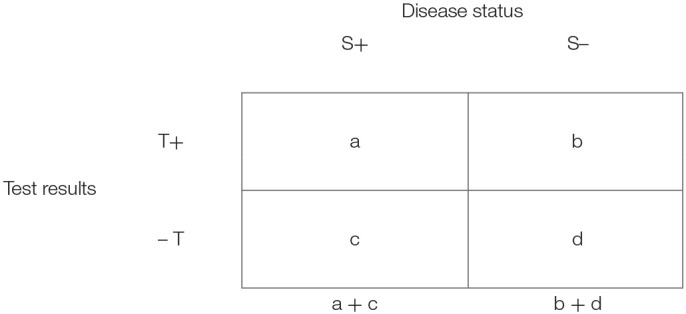
$sensitivity=P\left(T+|S+\right)=\frac{a}{a+c}$, $specificity=P\left(T-|S-\right)=\frac{d}{b+d}$
$Positive\;Likelihood\;Ratio=plr=\frac{sensitivity}{1-specificity}$
$Negative\;Likelihood\;Ratio=nlr=\frac{1-sensitivity}{specificity}$
Error terms for the study population:
$False\;positive\;fraction=fpf=\frac{b}{b+d}$
$False\;negative\;fraction=fnf=\frac{c}{a+c}$

The positive likelihood ratio, *plr*, is the ratio of *sensitivity* to the *false positive fraction, fpf* (i.e., *1-specificity*). Obviously, when *plr* = 1, the test is useless: its detection will be equally correct among persons with the disease and persons without the disease, (*sensitivity* = *fpr*). When *plr* > *1*, the higher the value of *plr*, the more effective is the test for detecting correctly persons with the disease, and it is less likely to wrongly identify a healthy person as a person with the disease. When *plr* < *1*, the *sensitivity* is lower than the *fpf*, so that the test detects correctly persons with the disease less frequently than it incorrectly identifies a healthy person as a person with a disease; i.e., the test is more misleading than helpful in detecting a disease.

Similarly, the negative likelihood ratio, *nlr*, is the ratio of the *false negative fraction, fnf* (i.e., 1-*sensitivity*) to *specificity*. When *nlr* = 1, the test is useless: it will equally not detect the disease incorrectly among persons with the disease (and fail to detect the disease) and without the disease (*specificity* = *fnf*).

When *nlr* < 1, the test is more effective: it identifies correctly healthy persons more frequently than it detects incorrectly persons with a disease as being without it. When *nlr* > *1*, the test is more misleading than helpful in detecting the absence of a disease.

### Estimating accuracy of diagnosis of a disease in the patient population (Table 2)

The application of a diagnostic test to a patient (target) population utilizes a similar 2 × 2 table (Table 2). To evaluate the effectiveness of the application of a diagnostic test in the patient population, the investigator first observes the outcome, i.e., the test results, and obtains information about the study factor, i.e., the disease status.

**Table 2:** Data presentation in a clinical study setting in a target patient population assessing the diagnostic capability of a test.

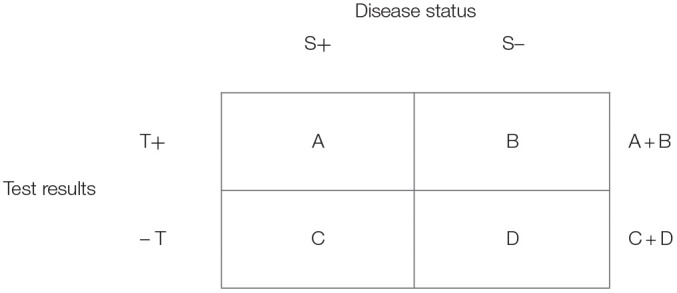
$PPV=P\left(S+|T+\right)=\frac{A}{A+B}$, $NPV=P\left(S-|T-\right)=\frac{D}{C+D}$
Positive Predictive Ratio = PPR = $\frac{\text{PPV}}{1-\text{NPV}}$
Negative Predictive Ratio = NPR = $\frac{1-\text{PPV}}{\text{NPV}}$
Error terms for the patient population:
False Positive Fraction = FPF = $\frac{\text{B}}{\text{A}+\text{B}}$ =1 − PPV
False Negative Fraction = FNF = $\frac{\text{C}}{\text{C}+\text{D}}$ = 1 − NPV

We use **upper-case letters** to describe screening in the patient population in a 2 × 2 table, Table 2. It is the data in this table that are of interest to the patient (and the physician), answering the following questions: When the test is positive, what is the probability that the patient has the disease? (Answerable by the PPV); and when the test is negative, what is the probability that the patient does not have the disease? (Answerable by the NPV).

We have suggested trustability ratios of a test, analogous to the likelihood ratios that have been discussed above (19).

The positive predictive ratio (PPR) is the ratio of the PPV to the False Negative Fraction in the patient population (FNF). Obviously, when PPR = 1 the test is useless: it will equally diagnose correctly (when the test is positive) and fail to diagnose persons with the disease (PPV = FNF).

In contrast, when PPR > 1, the higher the value of PPR, the more effective is the test for diagnosing correctly persons with the disease relative to diagnosing the absence of the disease when a person does have a disease, thus failing to yield a positive diagnosis. When PPR < 1, the test is misleading. It diagnoses unhealthy persons as not having a disease more frequently than it diagnoses correctly a person with a disease.

Similarly, the negative predictive ratio (NPR) is the ratio of the False Positive Fraction in the patient population (FPF) to the NPV. When NPR = 1, the test is useless: it will equally diagnose correctly persons without the disease (when the test is negative) and fail to diagnose persons without the disease (NPV = FPF). In contrast, when NPR < 1, the lower the value of NPR, the more effective is the test, as it correctly diagnoses healthy persons as persons without a disease more frequently than it diagnoses incorrectly a person without a disease as having the disease. When NPR > 1, the test is misleading. It diagnoses healthy persons as having a disease more frequently than it diagnoses correctly persons without a disease.

## Gain in certainty and summary measures

### Youden index as a summary measure of the detectability of a test in populations

In 1950, Youden proposed an index as a measure of the goodness of a test. Using the false positive and false negative fractions, the index is defined as

$$\begin{array}{l} J=1-\left(fpr+fnr\right)\\ \;=\left(1-fpr\right)+\left(1-fnr\right)-1=sensitivity+specificity-1 \end{array}$$

When *J* = 1, the test is always correct. In other words, there are no errors, so that *fpf* + *fnf* = 0; i.e., the test detects correctly the sickness status. When *J* = 0, assuming that *sensitivity* and *specificity* have equal importance in determining the expected gain, the above equation implies that when the *sensitivity* + *specificity* = 1, the test provides no overall information. In other words, the test is useless if the proportion of errors equals 100%, i.e., when the *fpf* + *fnf* = 1, *J* = 0. When *J* < 0 (between −1 and 0), the test is misleading; i.e., the tests results are negatively associated with the true diagnosis.

*J* can also be interpreted as the gained probability of correct detection information, i.e., the difference between the joint probabilities of correct detection (positive or negative detection), *sensitivity* ^*^ *specificity*, vs. the joint probabilities of incorrect detection, *fpf* ^*^ *fnf*.

$$\begin{array}{l} J=sensitivity*specificity-fpf*fnf\\ \;=sensitivity*specificity-\left(1-sensitivity\right)*\left(1-specificity\right)\\ \;=sensitivity+specificity-1 \end{array}$$

### Predictive summary index as a summary measure of diagnostability of a test in individuals (20)

We have proposed a summary index of the information, the Predictive Summary Index (PSI = Ψ) (17, Table 4), which is a measure of the additional information given by the test results, beyond the prior knowledge (the prevalence of the disease). Thus, the PSI summarizes the information in Table 2. Let us note that the information from a positive test result beyond what is already known a priori about the disease prevalence is PPV-Prevalence. Similarly, the information from a negative test result beyond what is already known a priori about the probability of no disease (the prevalence of no disease) is NPV-(1-Prevalence).

Thus, the overall information, i.e., the gain in certainty that we obtain after a test is performed, beyond what is already known, is calculable as a summary measure

$$\begin{array}{l} \text{Total gain in certainty}=\text{PPV}-\text{Prevalence}\\ \;+\text{NPV}-\left(1-\text{Prevalence}\right)\\ \;=\text{PPV}+\text{NPV}-1=\text{Ψ} \end{array}$$

Alternatively, Ψ is the information that is not derived from errors, FNF and FPF

$$\begin{array}{l} \text{Ψ}=1-\left(\text{FPF}+\text{FNF}\right)=1-\left[\left(1-\text{PPV}\right)+\left(1-\text{NPV}\right)\right]\\ \;=\text{PPV}+\text{NPV}-1 \end{array}$$

If Ψ = 1, the test is always correct, that is, there are no errors, so that FPF + FNF = 0; i.e., the test detects correctly the sickness status. When Ψ = 0, PPV + NPV = 1, and the test provides no overall information. In other words, the test is useless if the proportion of errors equals 100%, i.e., when FPF + FNF = 1, Ψ = 0. For example, if test results are random and the probability of both FPF and FNF is 50%, then the test is useless. When Ψ < 0, negative values (between −1 and 0) of Ψ make the test misleading; i.e., the tests results are negatively associated with the true diagnosis.

The PSI can also be interpreted as the gained probability of correct diagnosis information, i.e., the difference between the joint probabilities of correct diagnosis (positive or negative diagnosis) PPV^*^NPV vs. the joint probabilities of incorrect diagnosis FPF^*^FNP

$$\begin{array}{l} \text{PPV}*\text{NPV}-\text{FNR}*\text{FPR}\\ \;=\text{PPV}*\text{NPV}-\left[\left(1-\text{PPV}\right)*\left(1-\text{NPV}\right)\right]\\ \;=\text{PPV}*\text{NPV}-1+\text{NPV}+\text{PPV}-\text{NPV}*\text{PPV}=\psi \end{array}$$

## Utilization of bayes' theorem (1–20)

We have discussed the use of two different approaches for detection vs. diagnoses. In practice, the analyses are frequently performed in two stages: first one uses a selective (study) population for which the *sensitivity* and *specificity* are calculated, comparing a screening based on a test that is less accurate, costly, or invasive to a gold standard (GS), which may be a more accurate but more expensive or invasive test (e.g., biopsy), and then, Bayes' theorem and the prevalence are used, together with the *sensitivity* and *specificity*, to calculate the PPV or NPV. This is particularly useful when we do not have the information that is needed to construct Table 2; i.e., when we cannot calculate directly the PPV and NPV, because it is frequently unfeasible and unethical to perform both the diagnostic tests and an additional definitive test (the “gold standard” against which the test is evaluated) to determine the true diagnosis. Therefore, the test is evaluated in a selected sample of a population and only Table 1 can be constructed. PPV and NPV are then calculated from the *sensitivity* and *specificity* and the prevalence of the disease, applying Bayes' theorem.

## Discussion

Trevethan's article explains the difference between a “screening test” and a diagnostic test. We add and suggest that a distinction should be made between **detection (**in an already diagnosed population) and **diagnosis** (in the patient target population).

The term “screening” is used in two different situations. Screening can be performed in a **population** to detect a health condition for public health purposes. This activity is then evaluated in studies among persons with a known disease status, calculating the *sensitivity* and the *fnf* among persons with an already diagnosed disease and among those without a disease, calculating the *specificity* and the *fpf*. The prevalence of the disease in such studies is frequently artificial: the selection of participants with or without a disease may be dependent on the budget, availability of persons with known disease status (with or without a disease), and ethical considerations. Thus, the **predictive values cannot be calculated in these studies**. Such studies can help in the planning of health services in a public health setting and the possible use of the test for public health purposes (cost effectivity and utility).

In contrast, screening for a disease in the patient population in a clinical setting is performed for the purpose of diagnoses and treatments. This activity is then evaluated in studies among persons who present themselves at the clinics, without a known disease status. In fact, the clinical interaction focuses usually on the determination of a disease status. Screening in the clinical setting is thus performed for **diagnosis** purposes. In the clinical setting, it is possible to calculate the predictive values of a test, calculating the PPV and FPF among persons with a positive test result and the NPV and FNF among persons with negative test results. The prevalence of the disease in studies in the clinical setting is frequently the true prevalence in the population that is served by a clinic.

Further, we suggest that attention should be paid to ratio measures that are based on the *sensitivity* and *specificity*, that is, the likelihood ratios, *plr* and *nlr*. These measures can facilitate the assessment of a test in the public health setting. Similarly, we suggest that attention should be paid to ratio measures that are based on the PPV and NPV, i.e., the PPR and NPR above. These measures can facilitate the assessment of a test in the clinical setting and inform patients and physicians about the trustworthiness of a diagnostic test to diagnose a disease (the PPR) or a healthy status (the NPR).

We suggest that attention should be paid to summary measures, which indicate the gain in certainty in the **detection** of a disease in a public health setting and **diagnosis** of a disease in the patient population in the clinical setting, using measures such as the Youden index (*J*) and the PSI (ψ) (respectively).

We hope that our approach will clarify the methodology and the use of measures of detection in a screening setting vs. measures of diagnosis in a clinical setting.

## Author contributions

SL developed the algebraic expressions and theory. GG developed the concepts, the manuscript and reviewed the methodology, the paper, and conclusions.

### Conflict of interest statement

The authors declare that the research was conducted in the absence of any commercial or financial relationships that could be construed as a potential conflict of interest.

## References

[1] Trevethan R. Sensitivity, specificity, and predictive values: foundations, pliabilities, and pitfalls in research and practice. Front. Public Health (2017) 5:307. 10.3389/fpubh.2017.00307 29209603 PMC5701930

[2] Riffenburgh RH Statistics in Medicine. San Diego, CA: Academic Press (1999).

[3] Hirsch RP Riegelman RK Statistical Operations: Analysis of Health Research Data. Oxford: Blackwell Science (1996).

[4] Feinstein AR Principles of Medical Statistics. Boca Raton, FL: Chapman & Hall/CRC Press (2002).

[5] Weinstein MC Finberg HV. Clinical Decision Analysis. Philadelphia, PA: W. B. Saunders (1980).

[6] Altman DG Practical Statistics for Medical Research. London: Chapman & Hall (1991).

[7] Sackett DL Haynes RB Guyatt GH Tugwell P Clinical Epidemiology. 2nd ed. Boston, MA: Little Brown (1991).

[8] Kraemer HC Evaluation of Medical Tests: Objective and Quantitative Guidelines. London: Sage Publications (1992).

[9] Weiss NS Clinical Epidemiology: The Study of the Outcome Of Illness. Oxford: Oxford University Press (1996).

[10] Riegelman RK Studying a Study and Testing a Test: How to Read the Medical Evidence. 4th ed. Philadelphia, PA: Lippincott, Williams & Wilkins (2000).

[11] Knottnerus JA van Weel C General introduction: evaluation of diagnostic procedures. In: Knottnerus JA, editor. The Evidence Base of Clinical Diagnosis. London: BMJ Books (2002). p. 1–18.

[12] Sackett D Haynes RB. The architecture of diagnostic research. In: Knottnerus JA, editor. The Evidence Base of Clinical Diagnosis. London: BMJ Books (2002). p. 19–38. 10.1136/bmj.324.7336.539 PMC1122451 11872558

[13] Zhou XH Obuchowski NA McClish DK Statistical Methods in Diagnostic Medicine. New York, NY: Wiley-Interscience (2002).

[14] Pepe MS The Statistical Evaluation of Medical Tests for Classification and Prediction. Oxford Statistical Science Series 28. Oxford: Oxford University Press (2003).

[15] Morabia A (ed.). The History of Epidemiologic Methods and Concepts. Berlin: Birkhauser Verlag (2004).

[16] Rothman KJ Lash YL Greenland S Modern Epidemiology. 3rd ed. New York, NY: Lippincott Williams & Wilkins (2008).

[17] Grobbee DR Hoes AW Clinical Epidemiology. Burlington, MA: Jones and Bartlett Learning (2015).

[18] Linn S A new conceptual approach to teaching the interpretation of clinical tests. J Statistic Educ. (2004) 12 10.1080/10691898.2004.11910632

[19] Linn S. New patient-oriented diagnostic test characteristics analogous to the likelihood ratios conveyed information on trustworthiness. J Clin Epidemiol. (2005) 58:450–7. 10.1016/j.jclinepi.2004.07.009 15845331

[20] Linn S Grunau DP. New patient-oriented summary measure of net total gain in certainty for dichotomous diagnostic tests. EpidemiolPerspect Innovat. (2006) 3:11. 10.1186/1742-5573-3-11 17022816 PMC1635036

